# MicroRNA-125b regulates the expression of aggrecanase-1 (ADAMTS-4) in human osteoarthritic chondrocytes

**DOI:** 10.1186/ar4164

**Published:** 2013-02-13

**Authors:** Tetsuya Matsukawa, Tadahiro Sakai, Tomo Yonezawa, Hideki Hiraiwa, Takashi Hamada, Motoshige Nakashima, Yohei Ono, Shinya Ishizuka, Hiroyuki Nakahara, Martin K Lotz, Hiroshi Asahara, Naoki Ishiguro

**Affiliations:** 1 Department of Orthopaedic Surgery, Nagoya University Graduate School of Medicine, 65 Tsurumai-cho, Showa-ku, Nagoya 466-8550, Japan; 2 Department of Molecular and Experimental Medicine, The Scripps Research Institute, 10550 North Torrey Pines Road, La Jolla, CA 92037, USA; 3 Department of System Biomedicine, Tokyo Medical and Dental University, 1-5-45 Tsushima, Bunkyo-ku, Tokyo 113-8510, Japan; 4 Department of System Biomedicine, National Research Institute for Child Health and Development, 2-10-1 Okura, Setagaya-ku, Tokyo 157-8535, Japan

## Abstract

**Introduction:**

Increased expression of aggrecanase-1 (ADAMTS-4) has emerged as an important factor in osteoarthritis (OA) and other joint diseases. This study aimed to determine whether the expression of ADAMTS-4 in human chondrocytes is regulated by miRNA.

**Methods:**

MiRNA targets were identified using bioinformatics. Chondrocytes were isolated from knee cartilage and treated with interleukin-1 beta (IL-1β). Gene expression was quantified using TaqMan assays and protein production was determined by immunoblotting. Luciferase reporter assay was used to verify interaction between miRNA and target messenger RNA (mRNA).

**Results:**

*In silico *analysis predicted putative target sequence of miR-125b on ADAMTS-4. MiR-125b was expressed in both normal and OA chondrocytes, with significantly lower expression in OA chondrocytes than in normal chondrocytes. Furthermore, IL-1β-induced upregulation of ADAMTS-4 was suppressed by overexpression of miR-125b in human OA chondrocytes. In the luciferase reporter assay, mutation of the putative miR-125b binding site in the ADAMTS-4 3'UTR abrogated the suppressive effect of miR125.

**Conclusions:**

Our results indicate that miR-125b plays an important role in regulating the expression of ADAMTS-4 in human chondrocytes and this identifies miR-125b as a novel therapeutic target in OA.

## Introduction

Osteoarthritis (OA) is the most common form of arthritis and is characterized by loss of articular cartilage, which is ascribed mainly to proteolysis of the structural components of the extracellular matrix (ECM), that is, proteoglycans and collagens. Collagen degradation is principally carried out by several proteinases shedding an ectodomain of membrane proteins belonging to the matrix metalloproteinase (MMP) family, such as MMP1, MMP8, and MMP13 [1-3]. On the other hand, aggrecanases represent another class of proteinases belonging to the ADAMTS (a disintegrin and metalloproteinase with thrombospondin motifs) family. Biochemical studies indicate that ADAMTS 1, 4, 5, 8, 9, and 15 possess aggrecanase activity [4,5]. Song *et al. *[6] reported that knockdown of aggrecanase-1 (ADAMTS-4), aggrecanase-2 (ADAMTS-5), or both enzymes by small interfering RNA attenuates the degradation of aggrecan in human cartilage stimulated by a combination of both tumor necrosis factor-alpha (TNF-α) and oncostatin M. This suggests that both ADAMTS-4 and ADAMTS-5 contribute to aggrecanolysis in human tissue. Recent studies showed that ADAMTS-4 is selectively overexpressed in human OA cartilage, with a positive correlation with the degree of cartilage destruction, whereas ADAMTS-5 is similarly expressed in both normal and OA cartilages [7]. These results suggest that ADAMTS-4 is a major aggrecanase in human OA cartilage and its induction is involved in the pathogenesis of OA.

Proinflammatory cytokines secreted from chondrocytes such as interleukin-1 beta (IL-1β) and TNF-α contribute to the progression of OA [8-10]. IL-1β induces a cascade of inflammatory and catabolic events in chondrocytes. It also changes chondrocyte anabolism by suppressing the synthesis of proteoglycans and collagens [9,11-13].

MicroRNAs (miRNAs), which belong to a class of short 19 to 23 nucleotide non-coding RNAs, function as posttranscriptional negative regulators by promoting messenger RNA (mRNA) degradation or repressing translation through complementarily binding target sequences in the 3'-untranslated regions (3'-UTRs) of specific mRNA targets [14-17]. Degradation of target mRNAs depends on near-perfect complementarity while for repression of translation partial complementarity is sufficient [18].

Recent studies have identified the miRNA expression profiles in human OA chondrocytes [19,20], but no consensus on a specific miRNA being the regulator of ADAMTS-4 expression has been reached. In this study, we use bioinformatics to predict putative target sequences for miR-125a, miR-125b and miR-4319 in human ADAMTS-4 mRNA. We demonstrate that expression of miR-125b, but not miR-125a and miR-4319, is significantly decreased in human OA chondrocytes compared to normal chondrocytes. This prompted us to validate whether ADAMTS-4 is regulated by miR-125b in human OA chondrocytes. We also established a reporter system using the 3'-UTR of human ADAMTS4 mRNA, and showed that this is targeted by miR-125b.

## Materials and methods

### Specimen selection

Cartilage tissues were aseptically obtained at the time of total knee replacement from OA patients diagnosed according to the American College of Rheumatology criteria for this disease. The patients with Kellgren-Lawrence OA grades II, III, and IV included five females and two males, with a mean age of 74.1 years (ranging from 68 to 82 years). Articular cartilage from the weight-bearing, fibrillated areas of the femoral condyle and tibial plateau was cut into small pieces with a scalpel. Cartilage samples were minced and digested at 37°C with trypsin-EDTA solution for 30 min, incubated with 3 mg/ml type II collagenase (Worthington Biochemical Co, Lakewood, NJ, USA) in Dulbecco's modified Eagle's medium (DMEM; Gibco-BRL, Life Technologies, Merelbeke, Belgium) for 18 h, filtered through a nylon mesh, and washed extensively. Next, the isolated chondrocytes were seeded in 75 cm^2 ^culture flasks and incubated in DMEM containing 10% fetal bovine serum (FBS), penicillin (100 U/ml), and streptomycin (100 mg/ml) at 37°C in an atmosphere of 5% CO2. At confluence, the cells were detached and seeded in 12-well plates at a density of 2×10^5^/well. These experiments were performed in accordance with a protocol approved by the Ethics Committee of Nagoya University and all patients gave written consent for the use of their tissues for this research. Normal human articular chondrocytes from knee joints including three males, with a mean age of 37.6 years (ranging from 34 to 45) were purchased from Takara Bio Inc. (Shiga, Japan). Cartilage tissues for the direct isolation of microRNA, were harvested from human knee joints including three male, with a mean age of 18.3 years (ranging from 17 to 19) for normal samples (OA grade I), one male and two females, with a mean age of 57.7 years (ranging from 54 to 65) for OA samples (OA grade IV) and two male and one female, with a mean age of 50.7 years (ranging from 47 to 55) for normal age-matched (aging) samples (OA grade I), which were obtained from the tissue banks with approval of the Scripps Human Subjects Committee. Joints were processed within 72 hours postmortem.

### Cell culture

Articular cartilage tissues were cut into small pieces less than 1 mm^3^, and subsequently digested with 1 mg/mL collagenase from *Clostridium histolyticum *(Sigma-Aldrich Co., Tokyo, Japan) at 37°C for 16 h with shaking. Isolated primary cultured human OA articular chondrocytes and 293T cells were cultured in DMEM containing 10% FBS and 1% penicillin/streptomycin (Gibco-BRL, Life Technologies, Grand Island, NY, USA) at 37°C in a humidified atmosphere of 5% CO_2_. The medium was changed every three days.

### Treatment with IL-1β and preparation of miRNA

Primary cultured human chondrocytes were serum-starved overnight and then treated with recombinant human IL-1β (5 ng/mL; PeproTech, Rocky Hill, NJ, USA) for the indicated periods of time. Total RNA containing miRNA was extracted using the mirVana miRNA isolation kit (Applied Biosystems, Life Technologies, Carlsbad, CA, USA). Cells were collected by Lysis/Binding Buffer including 1/10 volume of miRNA Homogenate Additive and then Acid-Phenol:Chloroform was added. After centrifugation, the aqueous phase was transferred to fresh tubes and, after addition of 1.25 volumes of 100% ethanol, applied to a filter cartridge. After washing the filter, total RNA was extracted with nuclease-free water. Total RNA of cartilage tissue was isolated from fresh-frozen cartilage by homogenizing the tissue in a freezer mill (Spex, Metuchen, NJ, USA) and extracting the homogenate in TRIzol (Invitrogen, Life Technologies, Carlsbad, CA, USA).

### Transfection

MiRNA precursor (pre-miR-125b), antagomir (anti-miR-125b), or negative control oligonucleotides were obtained from Applied Biosystems. Primary cultured human chondrocytes were transfected with each oligonucleotides using Lipofectamine 2000 (Invitrogen, Life Technologies). Forty-eight hours after transfection, the cells were serum-starved overnight and then cultured in serum-free DMEM in the presence or absence of recombinant human IL-1β (5 ng/mL; PeproTech) for 24 hours. Cells were harvested and subjected to total RNA and protein extraction. HEK 293T cells were transfected with 1 μg reporter plasmid, 1 μg effector plasmid or point mutant of pre-miR-125b plasmid or negative control plasmid, and 10 ng of Renilla luciferase control vector using FuGENE HD Transfection Reagent (Promega, Madison, WI, USA) in 24-well plates. The detail of all constructs is described in 'Luciferase reporter assay'.

### Reverse transcription

Total RNA concentration was determined using absorption spectrochemical analysis. Ten nanograms of total RNA containing miRNA from control and experimental samples were reverse-transcribed using miR-125b stem-loop RT primer, 10× RT buffer, 100 mM deoxynucleoside triphosphates (dNTPs), 50 units of MultiScribe reverse transcriptase, and 20 units of RNase inhibitor at 16°C for 30 min, 42°C for 30 min, and 85°C for 5 min, and then the miRNA product was maintained at 4°C. Complementary DNA (cDNA) was produced using a High Capacity cDNA Reverse Transcription Kit (Applied Biosystems). Reverse transcription of cDNA from control and experimental samples was performed in 2 μL of 10× Reverse Transcription Buffer, 0.8 μL of 25× dNTP, 2 μL of 10× Random Primers, 1 μL of 50 units of MultiScribe Reverse Transcriptase, and 1 μL of RNase inhibitor at 25°C for 10 min, 37°C for 120 min, and 85°C for 5 s, and then the cDNA product was maintained at 4°C.

### Quantitative RT-PCR analysis of ADAMTS-4, ADAMTS-5, and miR-125b

Expression of ADAMTS-4, ADAMTS-5, glyceraldehyde 3-phosphate dehydrogenase (GAPDH) mRNA, mature miR-125a, miR-125b, miR-4319 or U6 small nuclear RNA (RNU6B) was determined using the TaqMan Gene Expression Assay (Applied Biosystems). The reactions for miRNA were performed in 20 μL of final volume containing 1.33 μL of RT product, 10 μL of 2× TaqMan Universal Master Mix, 1 μL of 20× Custom TaqMan Small RNA Assay, and 7.67 μL of nuclease-free water. The reactions for mRNA were performed in 20 μL of final volume containing 9 μL of RT product, 10 μL of 2× TaqMan Universal Master Mix, and 1 μL of 20× TaqMan Assay. To reduce variability among replicates, PCR premixes, which contain all reagents except for the template, were prepared and aliquotted into 0.2 ml thin well plates. The samples were incubated at 95°C for 10 min, followed by 40 cycles at 95°C for 30 s and 60°C for 1 min. Expression of GAPDH and RNU6B were used as internal controls to normalize each mRNA and miRNA expression, respectively. A threshold cycle was observed in the exponential phase of amplification, and quantification of relative expression levels was determined by the ΔΔC_t _method. The value of each control sample was set at 1 and was used to calculate the fold change in target genes.

### Immunoblotting

Cell lysates were prepared using RIPA buffer (sc-24948; Santa Cruz Biotechnology, Santa Cruz, CA, USA), subjected to sodium dodecyl sulfate-polyacrylamide gel electrophoresis (SDS-PAGE), and then transferred to nitrocellulose membranes (Bio-Rad, Hercules, CA, USA). Membranes were blocked with 5% nonfat dry milk in Tris-buffered saline containing 0.1% Tween 20 and then treated with diluted (1:1,000) polyclonal antibodies specific for ADAMTS-4 (Cosmo Bio Co. Ltd., Tokyo, Japan) or β actin (Cell Signaling Technology, Danvers, MA, USA). Immunoreactive proteins were visualized using horseradish peroxidase-conjugated secondary antibodies and enhanced chemiluminescence (Thermo Scientific, Tokyo, Japan). Images were captured using AE-9150 Ez-Capture II (Atto, Tokyo, Japan) and analyzed using CS Analyzer version 3.0 (Atto). Each band was scanned with background correction, and values were averaged and expressed as the mean ± standard deviation (SD).

### Luciferase reporter assay

The luciferase Vector pLuc2 was used to construct a reporter plasmid containing 1,389 base pairs of the 3'-UTR of human ADAMTS-4 mRNA (uc001fyu.2 obtained from UCSC genome browser [21]) containing two parts of the predicted complementary seed sequence for miR-125b (sequence of mature form: UCCCUGAGACCCUAACUUGUGA). Total RNA (1 μg) from human chondrocytes was reverse-transcribed into cDNA, and the ADAMTS-4 3′-UTR was amplified using primers (Forward;AAGATATCGGGGAGAACCCACAGGGAGACC and Reverse;TTGGTACCGAGTGTTATGCTAGTTCTTTATTTACATTATTTAATCCTCAC). PCR products and vectors were digested with EcoRV and Kpn I restriction endonucleases (New England Biolabs, Ipswich, MA, USA), digested vectors were dephosphorylated by calf intestinal phosphatase (CIP) and ligated 3′ to the luciferase reporter gene using the EcoRV and Kpn I site to generate pLuc2-ADAMTS-4 plasmid (reporter plasmid). We found two putative miR-125b sequences in the human genome, termed pre-miR-125b-1 (encoded on chromosomes 11) (uc010rzr.1) and pre-miR-125b-2 (encoded on chromosomes 21) (uc002ykf.3), respectively. Genomic sequences containing precursors of miR-125b were amplified from DNA from human chondrocytes using primers (miR125b-1 Forward;AAGGATCCCTTAGAGAAGAAATACCATACCACCTGTTTGTTGC and Reverse;TTGATATCACCTCAGACAAACTTTCTTTTCTTTTGTTTTTGCTTTAAAG, miR125b-2 Forward;AAGGATCCTTTCTACTGAAGTATTTTAAATAGTATTTAGAGGTAAAAGTCTAAGTG and Reverse;TTGATATCCTGATGATAAAGAAAAGCATTGTTCTTTTCTCCTAGGC). Each pre-miR cassette was digested with BamH I and EcoRV restriction endonucleases (New England Biolabs). Digested vectors were dephosphorylated by CIP and ligated into the pcDNA3.1 vector using the BamH I and EcoRV site to generate pcDNA3.1-pre-miR-125b-1 or -2 plasmid (effector plasmid). Using site-directed mutagenesis, we inserted several mutations into the seed sequence of miR-125b-1, miR125b-2 and ADAMTS-4 3′-UTR. Mutated vectors of miR-125b precursors were amplified from each effector plasmid template using primers (miR-125b-1 mutation1 Forward;AGAAAACATTGTTGCGCTCCTCTCAGt**gGG**tg**TC**ACCCTAACTTGTGATGTTTACCG and Reverse;CGGTAAACATCACAAGTTAGGGTGACACCCACTGAGAGGAGCGCAACAATGTTTTCT, miR-125b-1 mutation2 Forward;ACCGTTTAAATCCACGGGTTAGGCt**GA**tg**CC**acCTGCGAGTCGTGCTTTTGCATCCT and Reverse;AGGATGCAAAAGCACGACTCGCAGGTGGCATCAGCCTAACCCGTGGATTTAAACGGT, miR-125b-2 mutation1 Forward;TCTACCGCATCAAACCAGACTTTTCCTAGt**gGG**tg**TC**ACCCTAACTTGTGAGGTATTTTAGTAA and Reverse;TTACTAAAATACCTCACAAGTTAGGGTGACACCCACTAGGAAAAGTCTGGTTTGATGCGGTAGA, miR-125b-2 mutation2 Forward;AGGTATTTTAGTAACATCACAAGTCAGGCt**GA**tg**CC**acCTAGGCGGAGGGGAACCAGCAGCTTTG and Reverse;CAAAGCTGCTGGTTCCCCTCCGCCTAGGTGGCATCAGCCTGACTTGTGATGTTACTAAAATACCT; the bold letters indicate mutations. the uppercase letters indicate complementary binding in the precursor). Mutated vectors of ADAMTS-4 3′-UTR were amplified from reporter plasmid template using primers (ADAMTS-4 3′-UTR point mutation 1 Forward;GTCAAGGGTAGGGTGGGCCtt**G**a**CCC**aGTGAGGGATTATCT and Reverse;AGATAATCCCTCACTGGGTCAAGGCCCACCCTACCCTTGAC, ADAMTS-4 3′-UTR point mutation 2 Forward; GAACTCCTGACC**AG**a**CC**taATCGACCTGCCT and Reverse; AGGCAGGTCGATTAGGTCTGGTCAGGAGTTC; the bold letters indicate mutations. the uppercase letters indicate complementary binding in the ADAMTS-4 3′-UTR). After DpnI digestion, digested vectors were transformed, and then single colonies were picked from ampicillin agar plates, and plasmid DNA was prepared using a Plasmid Miniprep Kit (Qiagen, Valencia, CA, USA). Finally, we confirmed the sequence of established constructs as described above. The luciferase activity assay was performed 24 hours after transfection, using the Dual-Luciferase Reporter Assay System (Promega) and a VICTOR *X *Light Luminescence Plate Reader (PerkinElmer, Waltham, MA, USA). Firefly luciferase activity was normalized to Renilla luciferase activity. Each experiment was performed three times in triplicate

### Statistical analysis

All data were obtained from at least three independent experiments performed in triplicate. Statistical significance was determined using Student's *t *test. *P *values less than 0.05 were considered significant, and *P *values less than 0.001 were considered highly significant.

## Results

### Prediction of three miRNA target sequences in the 3'-UTR of human ADAMTS-4 mRNA

**Figure 1:**
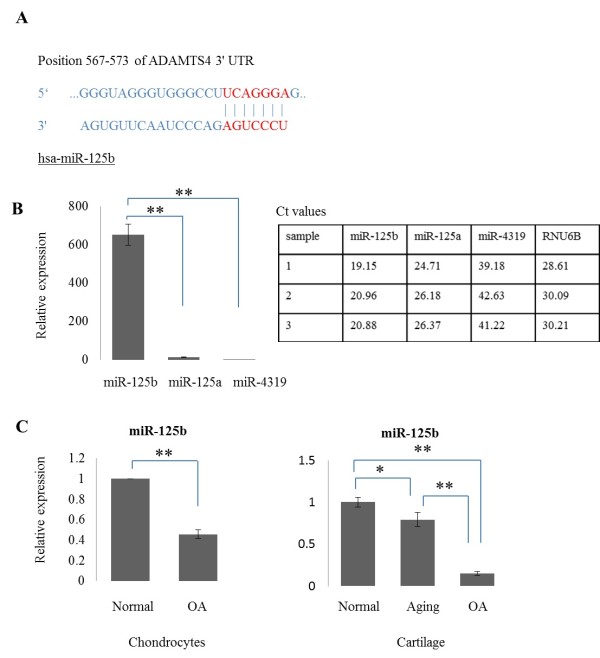
**Presence of miR-125b target sequence in the 3′-UTR of human ADAMTS-4 mRNA and its expression in primary cultured human chondrocytes and human cartilage tissues**. **(A) **TargetScan 6.2 predicted duplex of hsa-miR-125b seed sequence with target sequence in the 3'-UTR of human ADAMTS-4 mRNA. **(B) **Relative expression of miR-125b, miR125a and miR-4319 in normal chondrocytes (*n *= 3) and each threshold cycle (Ct) values from real-time PCR. **(C) **Relative expression of miR-125b in normal and osteoarthritic (OA) chondrocytes and normal, aging and OA cartilage tissue samples (*n *= 3 for normal chondrocytes normal, aging and OA cartilage from three different donors, *n *= 7 for OA chondrocytes from seven different donors). The data are expressed as mean and SEM of three independent experiments, each of which was run in triplicate. ** = *P *<0.001 as measured using an unpaired Student's *t *test. ADAMTS-4, aggrecanase-1; PCR, polymerase chain reaction; SEM, standard error of the mean; 3'-UTR, 3'-untranslated region.

We used TargetScan 6.2 [22] to identify the miRNAs that target ADAMTS-4 mRNA. TargetScan 6.2 identified a sequence conserved in the 3'-UTR of ADAMTS-4 mRNA that was complementary to the miR-125b (Figure 1A), miR-125a and miR-4319 seed sequences (data not shown). We hypothesized that any of these miRNAs, if expressed in human chondrocytes may play an important role in regulating ADAMTS-4 expression.

### MiR-125b expression in normal and OA chondrocytes

We determined the expression of miR-125b, miR-125a and miR-4319 using quantitative real-time PCR in normal and OA chondrocytes and cartilage tissues. In normal chondrocytes, miR-125b was strongly expressed while miR-125a and -4319 were barely detectable (Figure 1B). The expression of miR-125b was 49-fold higher than miR-125a and approximately two million-fold higher than miR-4319 (Figure 1B). Expression of miR-125b, but not miR-125a and -4319, was found to be 54% lower in OA chondrocytes compared to normal chondrocytes (Figure 1C and data not shown). Compared to young normal cartilage tissue samples (mean age = 18.3 years), the expression of miR-125b was 21% lower in normal cartilage tissue samples from older donors (mean age = 50.7 years) and 85% lower in age-matched OA cartilage tissue samples (mean age = 57.7 years) (Figure 1C).

### IL-1β induces ADAMTS-4 expression and suppresses miR-125b expression

**Figure 2:**
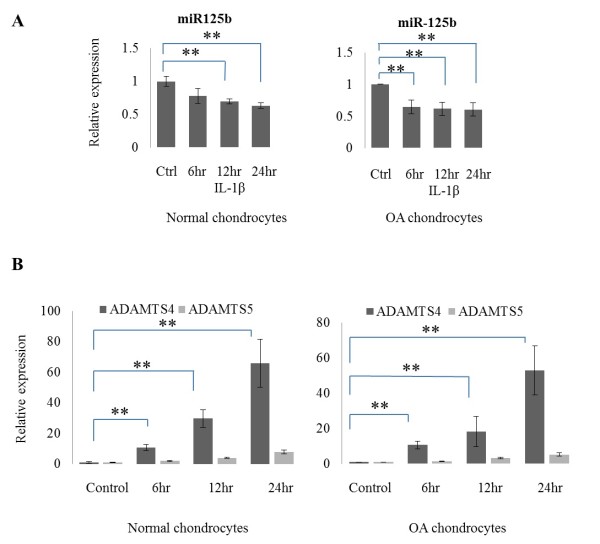
**IL-1**β **induces ADAMTS-4 expression and suppresses miR-125b expression**. **(A) **Relative expression of miR-125b in normal (*n *= 3) and osteoarthritic **(**OA) (*n *= 7) chondrocytes stimulated with IL-1β for 24 hours. **(B) **Kinetics of expression of mRNA of ADAMTS-4 and ADAMTS-5 in normal (*n *= 3) and OA (*n *= 7) chondrocytes treated with IL-1β for 6, 12 and 24 hours. The data are expressed as mean and SEM of three independent experiments, each of which was run in triplicate. ** = *P *<0.001 as measured using an unpaired Student's *t *test. ADAMTS-4, aggrecanase-1; SEM, standard error of the mean.

We examined whether IL-1β affects the expression of ADAMTS-4, ADAMTS-5 and miR-125b. IL-1β treatment suppressed miR-125b expression at 12 and 24 h in normal chondrocytes and at 6,12 and 24 h in OA chondrocytes (*P *<0.001) (Figure 2A). IL-1β treatment induced ADAMTS-4 expression in a time-dependent manner in both normal and OA chondrocytes (*P *<0.001). ADAMTS-5 mRNA expression was also increased in a time-dependent manner, but the magnitude of induction was much lower than for ADAMTS-4 mRNA (Figure 2B).

### Regulation of ADAMTS4 mRNA expression by miR-125b in human chondrocytes

**Figure 3:**
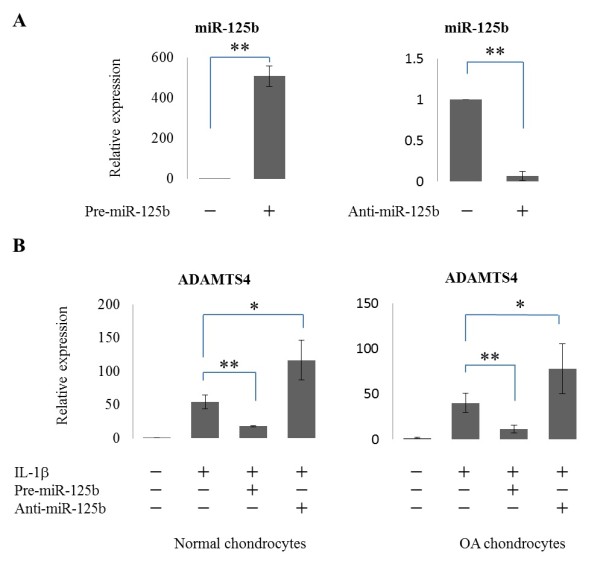
**Modulation of miR-125b affects ADAMTS-4 expression in OA chondrocytes**. **(A) **Relative expression level of miR-125b in human osteoarthritic (OA) chondrocytes transfected with pre-miR-125b, anti-miR-125b or negative control (*n *= 5). **(B) **Relative expression level of ADAMTS-4 mRNA 24 hours after IL-1β treatment in normal (*n *= 3) and OA (*n *= 5) chondrocytes transfected with pre-miR-125b, anti-miR-125b or negative control. The data are expressed as mean and SEM of three independent experiments, each of which was run in triplicate. ** = *P *<0.001, * = *P *<0.05 as measured using an unpaired Student's *t *test. ADAMTS-4, aggrecanase-1; anti-miR-125b, MiRNA-125b antagomir; pre-miR-125b MiRNA-125b precursor; SEM, standard error of the mean

To examine whether miR-125b regulates ADAMTS-4 in human normal and OA chondrocytes, we transfected the cells with pre-miR-125b or anti-miR-125b and treated with IL-1β. Significant induction of miR-125b was observed in cells transfected with pre-miR-125b compared to negative control (*P *<0.001), and suppression of miR-125b was observed in cells transfected with anti-miR-125b compared to negative control (*P *<0.001) (Figure 3A). MiR-125b decreased IL-1β-induced ADAMTS-4 mRNA (*P *<0.001) (Figure 3B). On the other hand, anti-miR-125b increased IL-1β-induced ADAMTS-4 mRNA (*P *<0.05) (Figure 3B).

### Regulation of ADAMTS-4 protein by miR-125b in human chondrocytes

**Figure 4:**
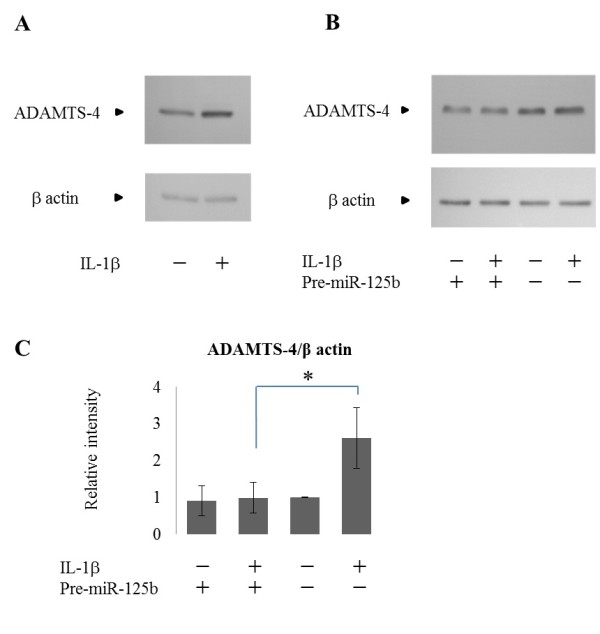
**MiR-125b suppresses ADAMTS-4 protein in OA chondrocytes**. **(A) **Increased production of ADAMTS-4 protein 24 hours after IL-1β treatment. **(B) **Osteoarthritic **(**OA) chondrocytes transfected with miR-125b or negative control incubated with or without IL-1β for 24 hours. **(C) **Bar graphs represent the average-fold increase ± SD in pixel intensity of bands representing ADAMTS-4 normalized to β actin, obtained from five separate western blot analyses for each cell condition. * = *P *<0.05 as measured using an unpaired Student's *t *test. ADAMTS-4, aggrecanase-1; IL-1 β, interleukin-1 beta; SD, standard deviation.

To examine whether miR-125b also regulates ADAMTS-4 protein levels, we performed western blotting on lysates from human OA chondrocytes transfected with miR-125b with or without IL-1β. In response to IL-1β, ADAMTS-4 protein was increased (Figure 4A). In cells transfected with miR-125b, ADAMTS-4 protein was reduced by approximately 62% compared to cells transfected with control miRNA (*P *<0.05) (Figure 4B and C).

### Functional validation of miR-125b using ADAMTS-4 3'-UTR luciferase reporter system

**Figure 5:**
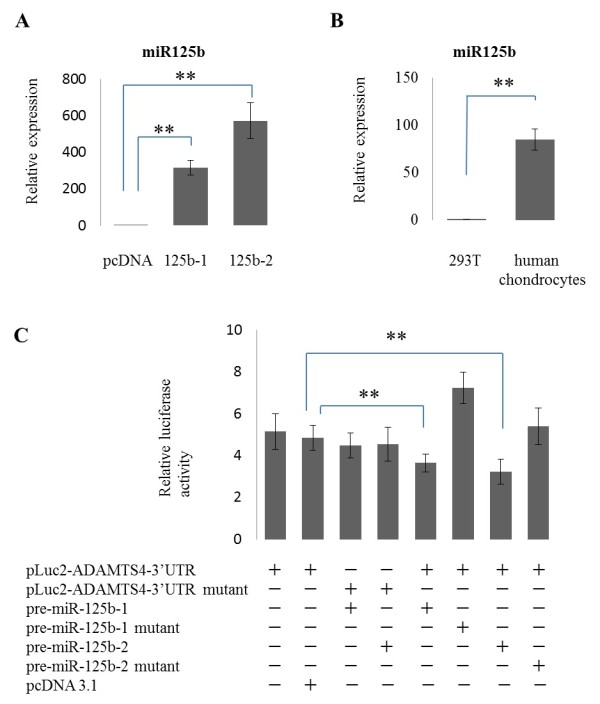
**Functional validation of miR-125b binding the putative target sequence in the 3′-UTR of ADAMTS-4 mRNA**. **(A) **Relative expression level of miR-125b in HEK293T cells transfected with pcDNA3.1-pre-miR-125b-1, -2 or negative control (*n *= 3). **(B) **Relative expression of miR-125b in 293T cells and normal chondrocytes (*n *= 3). **(C) **Luciferase activity in 293T cells co-transfected with pLuc2-ADAMTS-4 3'-UTR or -ADAMTS-4 3'-UTR mutant and pcDNA3.1-pre-miR-125b-1, -2, -125b-1 mutant, -125b-2 mutant or pcDNA3.1 (*n *= 3). The data are expressed as mean and SEM of three independent experiments, each of which was run in triplicate. ** = *P *<0.001 as measured using an unpaired Student's *t *test. ADAMTS-4, aggrecanase-1; pre-miR-125b MiRNA-125b precursor; SEM, standard error of the mean; 3'-UTR, 3'-untranslated region.

To confirm that miR-125b regulates ADAMTS-4 mRNA via directly binding to the predicted target sequence, we established a reporter system by combining luciferase and the 3'-UTR of human ADAMTS-4 mRNA. As we identified two putative precursors of miR-125b as described in Materials and Methods, we validated both in this assay. Significant induction of miR-125b was confirmed in cells transfected with two putative precursors of miR-125b compared to negative control (*P *<0.001) (Figure 5A). Compared to chondrocytes, 293T cells express very low levels of miR-125b (Figure 5B). We transiently co-transfected the ADAMTS-4 3'-UTR reporter construct or mutant reporter construct, and each pre-miR-125b or each mutant construct. The overexpression of miR-125b-1 or -2 markedly reduced luciferase activity compared to controls (*P *<0.001) (Figure 5C). We further confirmed these findings by using mutated forms of the 3'-UTR of human ADAMTS-4 mRNA and each miR-125b precursor. Transfection with the mutants abolished the suppression of luciferase activity (Figure 5C). These results indicate that suppression is due to direct miR-125b binding to complementary seed sequence in ADAMTS-4 3'-UTR.

## Discussion

In this study, we for the first time identified complementary sequence to miR-125b seed sequence in 3'-UTR of human ADAMTS-4 mRNA. Our results show that miR-125b but not miR-125a is strongly expressed in normal human chondrocytes and cartilage tissues. Both miR-125a and miR-125b were previously reported to be highly expressed in mouse spinal cord and liver, but only miR-125b was detected in other tissues [23]. In humans, there are two miR-125b gene loci: miR-125b-1 on chromosome 11q23 and miR-125b-2 on chromosome 21q21, which are differentially expressed in a tissue-specific pattern. Although their pre-microRNAs have slightly different sequences, their mature sequences are identical [24].

The present findings demonstrate that ADAMTS-4 is indeed regulated by miR-125b via direct binding the predicted target sequence, resulting in suppression of ADAMTS-4 at both mRNA and protein level. The potential interaction between miR-125b and human ADAMTS-4 mRNA was confirmed using luciferase reporter assay. In general, an 8 base-pair match is considered to represent a full-match seed sequence. For miR-125b and ADAMTS4 there is a 7 base-pair match, indicating partial complementarity. Computational and experimental evidence indicates that miRNA target sites with as little as 7 base pairs of complementarity to the miRNA 5' end are sufficient to confer regulation *in vivo *[25].

IL-1β is an important regulator of joint inflammation and cartilage degradation in OA and the present findings provide a link between IL-1β and miR-125b as chondrocyte treatment with IL-1β suppresses miR-125b expression, while inducing ADAMTS-4 expression. IL-1β has also been reported to induce ADAMTS-4 mRNA levels with a dependency on NF-κB signaling [26]. In addition to this pathway, miR-125b might be another factor to regulate ADAMTS-4 mRNA expression in response to IL-1β signaling. Furthermore, we demonstrated that in primary human OA chondrocytes, overexpression of miR-125b leads to a significant reduction in IL-1β- induced ADAMTS-4 production. Collectively, the results indicate that miR-125b directly regulates ADAMTS-4 in the context of activation by IL-1β as a key mediator of OA pathogenesis.

In this study, miR-125 suppressed ADAMTS-4 mRNA expression by 72% and protein production by 62% following IL-1β stimulation. In our previous study, miR-140 suppressed ADAMTS-5 mRNA expression by similar degree with IL-1β stimulation [27]. It thus appears that the two main aggrecanases in cartilage are regulated by different miRNAs.

We also predicted target sequence of miR-125a and miR-4319 in 3'-UTR ADAMTS-4 mRNA. However, our results indicate that miR-125a and miR-4319 expression in chondrocytes is very low compared to miR-125b and there are no significant changes in OA chondrocytes (data not shown). Thus, miR-125b, but not miR-125a and miR-4319, may play a role in OA development. The expression of miR-125b was reduced with a dependency on age, but the expression of miR-125b more significantly decreased in age-matched OA cartilage tissues compared to young normal cartilage tissues. The expression of miR-125b seems to be influenced by age-related factors, but more strongly by mechanisms related to OA.

The protective role of miR-125b in cartilage may extend beyond the suppression of ADAMTS-4. In other cell types miR-125b also suppressed MMP-13 and vascular endothelial cadherin [28,29]. MMP-13 is an important collagenase in articular cartilage [30], and vascular endothelial cadherin regulates angiogenesis, which is increased in OA-affected joints [31].

## Conclusions

This is the first report identifying a miRNA, miR-125b, as a posttranscriptional regulator of ADAMTS-4 in human chondrocytes. We have demonstrated the two precursors of miR-125b in human genome suppress the expression of ADAMTS-4 mRNA and protein. We suggest that to controlling the expression of miR-125b has potential as a novel approach for prevention and treatment of OA.

## Abbreviations

ADAMTS: a disintegrin and metalloproteinase with thrombospondin motifs; ADAMTS-4: aggrecanase-1; ADAMTS-5: aggrecanase-2; anti-miR-125b: MiRNA-125b antagomir; cDNA: complementary DNA; CIP: calf intestinal phosphatase; DMEM: Dulbecco's modified Eagle's medium; dNTP: deoxynucleoside triphosphate; ECM: extracellular matrix; FBS: fetal bovine serum; GAPDH: glyceraldehyde 3-phosphate dehydrogenase; IL-1β: interleukin-1 beta; MiRNA: microRNA; MMP: matrix metalloproteinase; mRNA: messenger RNA; OA: osteoarthritis; pre-miR-125b: MiRNA-125b precursor; RNU6B: U6 small nuclear RNA; TNF-α: tumor necrosis factor-alpha; 3'-UTRs: 3'-untranslated regions.

## Competing interests

The authors have declared that they have no competing interests.

## Authors' contributions

The study was designed by TS. TM and TY contributed to the acquisition of the data, analysis and interpretation, and drafting of the manuscript. TS, MKL, HA and NI contributed to the conception and interpretation of the article and the obtaining of funds for this study. HN, HH, TH, MN, YO, SI contributed to the samples collection, acquisition of the data, analysis and interpretation of the manuscript. All authors contributed to the revising of the manuscript and approved the final manuscript.
